# The regulatory effects of second-generation antipsychotics on lipid metabolism: Potential mechanisms mediated by the gut microbiota and therapeutic implications

**DOI:** 10.3389/fphar.2023.1097284

**Published:** 2023-01-25

**Authors:** Hui Chen, Ting Cao, Bikui Zhang, Hualin Cai

**Affiliations:** ^1^ Department of Pharmacy, The Second Xiangya Hospital, Central South University, Changsha, China; ^2^ Xiangya School of Pharmaceutical Sciences, Central South University, Changsha, China; ^3^ Institute of Clinical Pharmacy, Central South University, Changsha, China; ^4^ International Research Center for Precision Medicine, Transformative Technology and Software Services, Changsha, Hunan, China

**Keywords:** gut microbiome, SGAs, lipid disturbances, gut microbiota-brain axis, SCFAs, BAs

## Abstract

Second-generation antipsychotics (SGAs) are the mainstay of treatment for schizophrenia and other neuropsychiatric diseases but cause a high risk of disruption to lipid metabolism, which is an intractable therapeutic challenge worldwide. Although the exact mechanisms underlying this lipid disturbance are complex, an increasing body of evidence has suggested the involvement of the gut microbiota in SGA-induced lipid dysregulation since SGA treatment may alter the abundance and composition of the intestinal microflora. The subsequent effects involve the generation of different categories of signaling molecules by gut microbes such as endogenous cannabinoids, cholesterol, short-chain fatty acids (SCFAs), bile acids (BAs), and gut hormones that regulate lipid metabolism. On the one hand, these signaling molecules can directly activate the vagus nerve or be transported into the brain to influence appetite *via* the gut–brain axis. On the other hand, these molecules can also regulate related lipid metabolism *via* peripheral signaling pathways. Interestingly, therapeutic strategies directly targeting the gut microbiota and related metabolites seem to have promising efficacy in the treatment of SGA-induced lipid disturbances. Thus, this review provides a comprehensive understanding of how SGAs can induce disturbances in lipid metabolism by altering the gut microbiota.

## Introduction

The use of antipsychotic medications as a treatment for patients with schizophrenia is surging, and the incidence of schizophrenia is also rising dramatically worldwide (Hert et al., 2011; Gonçalves et al., 2015). However, long-term use of these drugs can cause numerous adverse effects on patients, especially the disruption of lipid levels, including high-density lipoprotein (HDL), low-density lipoprotein (LDL), triglyceride (TG), and total cholesterol (TC) (Jaberi et al., 2020). Although individuals with schizophrenia may exhibit dyslipidemia before the initiation of treatment, mounting evidence has shown that antipsychotics can independently induce further abnormalities. It has been noted that patients with first-episode schizophrenia have abnormal lipid profiles, and those with multiple-episode schizophrenia are more likely to have dyslipidemia (Mackin et al., 2007; Vancampfort et al., 2015; Mhalla et al., 2018; Pillinger et al., 2019; Yang et al., 2022). Notably, second-generation antipsychotics (SGAs) have stronger associations with lipid abnormalities than first-generation antipsychotics (FGAs) have (Buhagiar and Jabbar, 2019). Metabolic abnormalities especially lipid metabolism disorders, are major risk factors contributing to cardiovascular events (Fan et al., 2013). They also play a role in the pathophysiological process of systemic organ damage and are causative factors in the development and progression of atherosclerotic cardiovascular disease. According to previous reports, patients with schizophrenia have a life expectancy that could be 15 years shorter than that of the general population. Additionally, more than two-thirds of patients with schizophrenia die from coronary heart disease, which is significantly higher than the mortality rate in the general population (Hennekens et al., 2005). A growing body of research indicated that SGA-induced disturbances of lipid metabolism and other metabolic abnormalities are the key factor linking to an increased risk of cardiovascular disease in patients with schizophrenia, in addition to some confounding risk factors such as smoking, physical inactivity, unhealthy lifestyle, and poor dietary habits (Arias et al., 2018).

Although the particular processes by which SGAs cause dysfunctional lipid metabolism are complex, a growing body of evidence suggests that the gut microbiota is involved in SGA-induced defects in lipid metabolism. From birth, humans have microbes in their digestive tract (Yatsunenko et al., 2012). Hundreds of millions of microorganisms, including bacteria, fungi, and viruses, exist in the healthy human gastrointestinal system, forming a microbial community that has a major impact on the body (Mirzaei and Maurice, 2017). A large number of these bacteria make up the collective intestinal flora. The intestinal flora contains 1000 to 1500 species of bacteria, which outnumber the body’s cells by more than 10 times (Kim and Jazwinski, 2018) and have more than 100 times the total number of genes as humans (Cox et al., 2019). These bacteria play important roles in host metabolism, digestion, the immune system, and the central nervous system (John and Mullin, 2016; Rogers et al., 2016; Dinan and Cryan, 2017; Ipci et al., 2017; Kanji et al., 2018). The theory that the gut microbiota affects lipid metabolism has been extensively studied in mice. For example, germ-free (GF) mice on a chow diet showed lower fasting systemic TG, TC, HDL cholesterol, and portal vein TG (Martinez-Guryn et al., 2018), as well as higher liver cholesterol and lower TG levels, than conventionally raised (Conv-R) mice (Rabot et al., 2010). Rabot et al. found that Conv-R mice had increased blood TG, HDL, and TC levels after consuming a high-fat diet (Rabot et al., 2010). To maintain the same weight as Conv-R mice, GF mice had to increase their caloric intake by at least 30% (Hsiao et al., 2008). Further evidence that the intestinal flora affects lipid metabolism has been observed in fecal transplantation experiments. Peter et al. showed for the 1 time that the ability of the gut microbiota to harvest energy from the diet was a transmissible trait. GF mice colonized with an ‟obesity microbiota” had a much higher increase in total fat than GF mice colonized with a ‟lean microbiota” (Turnbaugh et al., 2006). Similarly, obese patients with reduced microbial gene abundance (40%) showed more pronounced metabolic disturbances and had increased total serum cholesterol and serum TG levels (Cotillard et al., 2013).

The sex and age of the host as well as the site in the gastrointestinal tract influence the makeup and variety of the intestinal flora (Kim and Jazwinski, 2018; Cox et al., 2019). Independent of host variables, diet, lifestyle, and medicine can alter the composition of the gut flora (Kanji et al., 2018). Studies in recent years have shown that SGAs have some antibacterial activity and can alter the gut microbiota of patients with psychosis (Nehme et al., 2018; Ait Chait et al., 2020). Olanzapine can have direct antibacterial *in vitro* effects against the mammalian gut bacteria *Escherichia coli* and *Enterococcus faecalis*, which are the two most common species in the intestine (*E. coli*: Proteobacteria; *E. faecalis*: Firmicutes) (Morgan et al., 2014). Similarly, chlorpromazine (Kristiansen, 1979) has shown antibacterial effects against *Mycobacterium tuberculosis in vitro*, and thioridazine (Thorsing et al., 2013) acts against methicillin-resistant *Staphylococcus aureus*. These medications targeted a more comparable pattern of species than their degree of chemical similarity would suggest (Maier et al., 2018). This raises the possibility that direct bacterial inhibition by SGAs is not merely a side effect but also a part of their molecular mechanism.

**FIGURE 1:**
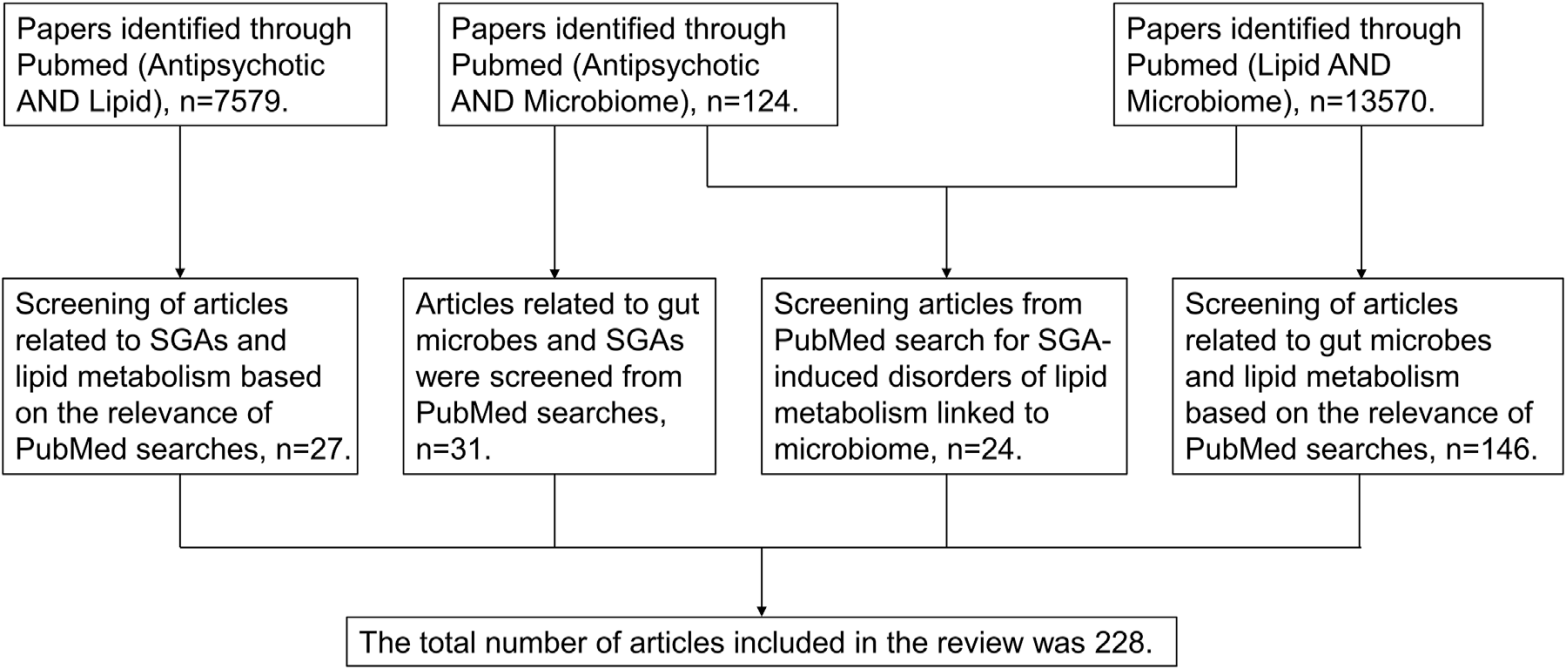
Flow chart diagram delineating search method.

The effect of gut microbes on lipid metabolism has been supported by many *in vivo* and *in vitro* studies, and evidence of the effect of SGAs on gut microbes is gradually emerging with the advancement of microbiological research techniques. Furthermore, positive results have been achieved with therapeutic strategies that directly target the gut microbiota and related metabolites, thereby ameliorating antipsychotic-induced disorders of lipid metabolism. This certainly identifies the gut microbiome as a potential target and establishes that the potential mechanism underlying lipid metabolism disturbances associated with antipsychotics is worthy of further investigation. However, the role of the gut microbiome in antipsychotic-induced disorders of lipid metabolism has not been systematically explained. This review aims to provide a comprehensive understanding of the potential for SGAs to alter the gut microbiota and promote adverse lipid metabolism events. Keyword search on PubMed is detailed in Figure 1, based on the Preferred Reporting Item Guidelines for Systematic Reviews and Meta-Analyses.

## The critical impact of SGAs on the gut microbiota: Evidence from animals and humans

Research on the microbiota in schizophrenia patients treated with SGAs is very scarce within the general microbiota literature. Several studies have investigated the effect of the gut microbiome on animal and human models (Table 1; Table 2). Mice treated with risperidone (Ridaura et al., 2013; Bahr et al., 2015b; Riedl et al., 2021) and olanzapine (Morgan et al., 2014) have increased ratios of Firmicutes to Bacteroidetes, which is one of the distinguishing features of the microbiota of obese individuals (Turnbaugh et al., 2006; Schwiertz et al., 2010; Ferrer et al., 2013). Firmicutes and Proteobacteria are the two major phyla associated with the human intestinal microbiome, constituting the majority of intestinal bacteria (approximately 90%) (Human Microbiome Project Consortium, 2012). These studies have been well replicated in humans (Bahr et al., 2015a; Yuan et al., 2018; Ma et al., 2020). Exceptions to this rule are Kao et al. (Kao et al., 2018) and Pelka et al. (Pełka-Wysiecka et al., 2019). These studies showed no significant effects of olanzapine on the gut microbiota in female rats or in women with schizophrenia. The results of studies on changes in the phylum Actinomycetes are also inconsistent. Bahr et al. (Bahr et al., 2015b) found an increase in the relative abundance of the actinomycete clade in the feces of mice treated with risperidone, whereas Davey et al. (Davey et al., 2012) showed that the relative abundance of the actinomycete clade in mice administered olanzapine was decreased. During olanzapine treatment, the relative abundance of Erysipelotrichi and Gammaproteobacteria increased, while the relative abundance of Bacteroidia decreased (Morgan et al., 2014). Both Erysipelotrichi and Gammaproteobacteria are associated with non-alcoholic fatty liver disease (NAFLD) independent of weight gain (Spencer et al., 2011; Henao-Mejia et al., 2012). Risperidone treatment increased the relative abundance of *Allobaculum* spp. *Bacteroides* spp. *Bifidobacterium* spp. and *E. coli* and decreased the relative abundance of *Lactobacillus* spp. *Alistipes* spp. *Akkermansia* spp, and *Clostridium coccoides* groups (Ridaura et al., 2013; Bahr et al., 2015b; Yuan et al., 2018). It should be noted that the results of studies on the change in relative abundance of *Bacteroides* spp. Are inconsistent: one study showed an increase, while another showed no significant change (Bahr et al., 2015b; Yuan et al., 2018). Furthermore, the abundance of *Bifidobacterium* spp. In the feces of mice treated with risperidone was negatively correlated with serum LDL levels; *E. coli* was negatively correlated with serum TG levels (Yuan et al., 2018). However, there is little evidence regarding the relationship between changes in lipid metabolism and alterations in the gut microbiota in humans following SGA treatment. Of particular interest is the search for potential probiotic bacteria such as *Akkermansia muciniphila*, which is a previously reported ‘lean gut microbiota’ species. *A. muciniphila*, a member of the phylum Verrucomicrobia, is the only species of the genus *Akkermansia*. *A. muciniphila* is a mucin degrader in the intestine and is significantly and negatively associated with altered fat metabolism and obesity (Henao-Mejia et al., 2012; Schneeberger et al., 2015). A significantly reduced abundance of fecal *A. muciniphila* was found in patients with bipolar disorder who were treated with a range of SGAs, such as clozapine, olanzapine, and risperidone, compared to controls (Flowers et al., 2017).

**TABLE 1:** Studies of SGAs and microbiota in rodents.

Subjects	Drugs	Composition	Body weight	Food intake	Key findings i: Adipose tissue ii: Liver iii: Plasma	References
Sprague Dawley rats	Olanzapine	↑: Firmicutes; ↓: Actinobacteria (significant in females), Proteobacteria, and Bacteroidetes	↑ (only in females)	↑ (mostly in females)	i: ↑ visceral fat; ↓ gene expression of SREBP-1c (in females); ↑ inflammation markers (IL-6 mRNA expression in females and 4-fold increase (insignificant) in males, CD68 expression in females and males); iii: ↓ circulating levels of ghrelin in females; ↑ hypothalamic expression of ghrelin 1a receptor mRNA in males; inflammation markers: ↑ IL-8 and IL-1β in females, ↓ IL-6 and TNFα in males	Davey et al. (2012)
Sprague Dawley rats	Olanzapine	↑: Firmicutes; ↓: Bacteroidetes	↑	↑	i: ↑ fat mass; ↑macrophage infiltration; ↑ inflammation markers (CD68 Mrna); ii: ↑ hepatic expression of FAS, SREBP-1c, and ACC iii: ↑ FFAs	Davey et al. (2013)
C57BL/6J mice	Olanzapine	↑: Erysipelotrichi, Actinobacteria, and Gammaproteobacteria; ↓: Bacteroidia	↑			Morgan et al. (2014)
C57BL/6J mice	Risperidone	↑phyla: Firmicutes and Actinobacteria; ↑genera: *Bacteroides*, Allobaculum, Turicibacter, and Aneroplasma; ↓phyla: Bacteroidetes and Proteobacteria; ↓ genera: Alistipes, *Lactobacillus*, and Akkermansia	↑			Bahr et al. (2015b)
Sprague Dawley rats	Olanzapine		↑		i: ↓ GPR43 mRNA; ii: ↑ hepatic expression of ACC mRNA; iii: ↑ acetate; inflammation markers: ↑ IL-1β and TNFα, ↓ IL-8	Kao et al. (2018)
Sprague Dawley rats	Aripiprazole	↑: Peptostreptococcaceae, Clostridiaceae, and Ruminococcaceae; ↓: Ruminococcus _1_			iii: ↑ acetate; ↑ isovalerate;	Cussotto et al. (2019)

SREBP-1c, sterol response element-binding protein-1c; CD68, Cluster of Differentiation 68; IL-6, interleukine-6; IL-8, interleukine-8; IL-1β, interleukine-1beta; TNF-α, tumor necrosis factor-alpha; FAS, fatty acid synthase; FFAs, Free fat acids; ACC, acetyl coenzyme A carboxylase; GPR43, G-protein-coupled receptor 43.

**TABLE 2:** Studies of SGAs and microbiota in humans.

Subjects	Drugs	Composition	Key findings	References
Male child with Mental Disorders	Risperidone	↓ Bacteroidetes than Firmicutes relative	i: Higher levels of KEGG-associated pathways for butyrate and propionate metabolism were found within the risperidone treatment group compared with psychiatric controls; ii: The microbiota of participants treated chronically with risperidone were enriched for KEGG orthologs affecting tryptophan metabolism	Bahr et al. (2015a)
Patients with Bipolar Disorder	Clozapine, olanzapine, risperidone, quetiapine, asenipine, ziprasodone, lurasidone, aripiprazole, paliperidone, and iloperidone	↑: Lachnospiraceae; ↓: Akkermansia and Sutterella	i: ↓ in species diversity for the SGA-treated cohort, a correlation that was stronger in SGA-treated females	Flowers et al. (2017)
Elderly (age ≥ 65) multimorbid (≥2 chronic diseases) patients	Multiple antipsychotics	strong associations with Prevotella; Desulfovibrionaceae family; Succinivibrionaceae family		Ticinesi et al. (2017)
Normal weight patients with first episode schizophrenia	Risperidone	↑: Bifidobacterium spp. And Escherichia coli; ↓: Clostridium coccoides group and *Lactobacillus* spp	i: It is speculated that the increased level of Bifidobacterium spp. could be a compensatory response to counteract weight gain and the upregulated inflammatory status; ii: Upregulated status of inflammation and oxidative stress	Yuan et al. (2018)
Patients with Mental Disorders	Clozapine, olanzapine, risperidone, quetiapine, or ziprasidone	↓: Alistipes	i: SGA-treated female patients exhibited less microbial diversity than those not treated with SGAs	Flowers et al. (2019)
Patients with Schizophrenia	Olanzapine	The microbiota in patients with the schizophrenia can be clustered into different taxonomical (Type 1, with a predominance of Prevotella, and Type 2 with a higher abundance of *Bacteroides*, Blautia, and Clostridium) and functional groups; the microbiota does not change during 6 weeks of treatment with olanzapine	i: Not associated with the weight gain that occurs in women treated. with olanzapine, as well as the treatment effectiveness; ii: Patients with schizophrenia were clustered at the level of KEGG genes, modules, and pathways	Pełka-Wysiecka et al. (2019)
Patients with Schizophrenia	Multiple antipsychotics	Both antipsychotic-naïve schizophrenics and Antipsychotic-experienced schizophrenics; ↑ family: Christensenellaceae and Enterobacteriaceae; ↑ genus: Escherichia from family Enterobacteriaceae; ↓ family: Turicibacteraceae and Pasteurellaceae. compared with Compare patients with antipsychotic-naïve schizophrenics, Antipsychotic-experienced schizophrenics: ↑ family: Peptostreptococcaceae and Veillonellaceae; ↑ genus: Megasphaera, *Fusobacterium* and SMB53		Ma et al. (2020)
Patients with Schizophrenia	Risperidone	↓: Lachnoclostridium; ↑: Rombutsia	i: Immune and inflammatory processes, such as increased levels of hs-CRP and HCY may be a compensatory response to counteract disruption of lipid metabolism	Yuan et al. (2021)
Patients with Schizophrenia	Amisulpride	↑: Dorea, Desulfovibrio, Butyricicoccus; ↓: *Actinomyces* and Porphyromonas	i: Increased IL-4 levels and decreased IL-6 levels could be a compensatory response to lipid metabolic disturbance; ii: Downregulation of butanoate metabolism might be a compensatory reaction to lipid metabolism dysfunction	Zheng et al. (2022)

KEGG, kyoto encyclopedia of genes and genomes; hs-CRP, high-sensitivity C-reactive protein; HCY, homocysteine; IL-4, interleukine-4; IL-6, interleukine-6.

## SGA-induced lipid disorders: An intimate involvement with microbiota

To describe the relationship between intestinal flora and SGA-induced lipid metabolism, Davey et al. investigated the effect of antibiotic-induced alterations in the gut microbiota on the metabolism of female rats treated with olanzapine (Davey et al., 2013). They found that clinically relevant doses of olanzapine accelerated metabolic disturbances and weight gain in C57BL/6J mice fed a high-fat diet. When the rats were treated with both olanzapine and a cocktail of broad-spectrum antibiotics, including oral neomycin, metronidazole, and polymyxin, the increases in the proportions of Firmicutes and Bacteroidetes bacteria were reversed, and this treatment reversed the olanzapine-induced metabolic disturbances and weight gain induced by high-fat diets in C57BL/6J mice. Thus, Morgan et al. conducted a further study and found that this phenomenon was consistent with a previously described study conducted under sterile conditions but that olanzapine-induced metabolic disturbances and weight gain occurred soon after gut microbial colonization (Morgan et al., 2014). Further experimental work has been conducted on mice treated with prebiotics in combination with SGAs. Coadministration of olanzapine and the prebiotic B-GOS led to a significant increase in circulating levels of TNFα in mice, which has been reported to affect lipid metabolism, elevate fecal *Bifidobacterium* spp. And reduce body weight, and these effects were not seen in response to olanzapine or B-GOS treatment alone (Kao et al., 2018). Similarly, the probiotic *A. muciniphila* was observed to have a similar effect (Huang et al., 2021). These studies suggest that intestinal microbes are necessary and sufficient for SGA-induced disruption of lipid metabolism. It is worth noting that none of these experiments were replicated in humans.

## Sex differences in SGA-induced lipid disorders: A potential role of microbiota?

Accumulating evidence shows that female patients who take SGAs seem to have poorer lipid profiles than those of male patients, as well as a higher prevalence of metabolic syndrome and cardiovascular risk factors, including weight gain and dyslipidemia (Chen et al., 2020). It is noteworthy that sex-dependent differences in the host’s metabolism may be associated with gut microbiota (Wu et al., 2007; Lange et al., 2017). For example, women usually have considerably higher Firmicutes:Bacteroidetes ratios as compared to men in a population-based cross-sectional investigation (Koliada et al., 2021). Given that SGA-induced lipid disturbances are frequently associated with an increased ratio of Firmicutes to Bacteroidetes, this finding raises the possibility that women are more susceptible than men to abnormal lipid metabolism (Morgan et al., 2014). Another substantial indication that men and women have different microbes is the fact that sex hormones can affect the composition of the host microbiome. Significant changes in the host gut microbiota, such as a drop in the abundance of butyrate-producing bacteria and a decline in alpha diversity, are linked to elevated levels of estrogen in pregnant women (Koren et al., 2012). These differentiations can result in a significant impact on SAG-induced changes in lipid metabolism between genders. Unfortunately, available studies are not enough to systematically explain the link between sex differences in gut microbes and sex differences in disorders of lipid metabolism caused by antipsychotics. However, this phenomenon might offer some guidance for future studies on sex differences regarding the side effects of SGAs.

## Mechanisms of SGA-induced disorders of lipid metabolism mediated by the intestinal microbiota

Microorganisms and their metabolites are crucial in understanding how the gut microbiome is implicated in SGA-induced systemic lipid disorders (Skonieczna-Żydecka et al., 2019). Short-chain fatty acids (SCFAs), bile acids (BAs), and neurotransmitters are among the metabolites that the intestinal microbiota can create. Bacteroidetes and Firmicutes can create butyric acid, which accounts for approximately 20% and 60% of the total intestinal flora, respectively, while Proteobacteria and Actinobacteria produce very small amounts of SCFAs (5%–10% and 3%, respectively). Sulfate-reducing bacteria may use lactic acid to make acetic acid and hydrogen sulfide, while Veillonellaceae can convert it to propionic acid. Bacteroidetes is a phylum that can convert succinic acid to propionic acid, and its population density is related to the amount of propionic acid in the intestine (Karlsson et al., 2013). The dominant genera for BA production are *Lactobacillus*, *Bifidobacterium*, *Enterobacter*, *Anaplasma*, and *Clostridium* (Krautkramer et al., 2021). In addition, *Candida*, *Streptococcus*, and *Escherichia* can produce 5-hydroxytryptamine (5-HT; serotonin) (Krautkramer et al., 2021). Approximately 36% of the small molecules in human blood are produced or modified by microbial metabolism. The total SCFA concentration in the colons of GF mice is 100 times higher than that of ordinary animals. Acetic acid is the most concentrated SCFA in organisms and is central to carbohydrate and lipid metabolic pathways (Kimura et al., 2020). Miller et al. used radioisotope analysis and showed that the main pathway for bacterial production of acetate is the Wood–Ljungdahl pathway (Miller and Wolin, 1996). Moreover, olanzapine treatment of patients with schizophrenia significantly increased plasma acetate concentrations (Kao et al., 2018). Increased levels of the Kyoto Encyclopedia of Genes and Genomes (KEGG) metabolic pathways of butyric acid and propionic acid were found in a group of schizophrenia patients treated with risperidone (Bahr et al., 2015a). Hepatocytes create primary BAs, which are then 7-dehydroxylated by intestinal bacteria to produce secondary BAs. The gut microbiome affects the composition of the BA pool, such as the primary BA/secondary BA ratio, and thus affects the function of BAs, especially the metabolism of lipids (Wahlström et al., 2016). SGAs can cause an increase in total serum BAs. Specific SGAs, including chlorpromazine (BREUER, 1965), olanzapine (Lui et al., 2009), haloperidol (Fuller et al., 1977), risperidone (Wright and Vandenberg, 2007), and quetiapine (Shpaner et al., 2008), have been reported to cause cholestasis in a small number of patients taking the medication and are unpredictable with no significant correlation between dose and the duration of administration.

The intestinal microbiota signals to enteroendocrine (EE) cells through metabolites in multiple ways, resulting in the secretion of a range of intestinal hormones, such as glucagon-like peptide 1 (GLP-1), 5-HT, gastrin, leptin, cholecystokinin (CCK) and peptide tyrosine-tyrosine (PYY) (Martin et al., 2019). First, the microbiota produces SCFAs, which signal to EE cells through free fatty acid receptors 2 or 3 (FFAR2/3) (Offermanns, 2014) or by activating nuclear histone deacetylase (HDAC) (Waldecker et al., 2008; Fellows et al., 2018; Larraufie et al., 2018). Second, secondary BAs signal to EE cells *via* the Takeda G-protein-coupled BA receptor TGR5 or the nuclear receptor known as farnesoid X receptor (FXR) (Wahlström et al., 2016). Numerous human and animal studies have demonstrated that leptin, ghrelin (Sentissi et al., 2008), and 5-HT levels (Bahr et al., 2015a) have a substantial positive link to aberrant lipid profiles and body mass index before and after SGA treatment in schizophrenia patients. This supports the idea that the intestinal flora and its metabolites play an important role in SGA-induced metabolic abnormalities.

### Specific microorganisms can synthesize specific lipids

There appear to be distinct bacteria that are more or less related to specific classes of lipids. Gut commensal microorganisms (*Bacteroides*, *Prevotella* and *Porphyromonas*) are significantly altered by SGAs, and they can produce sphingolipids, including ceramide phospholipids and deoxy sphingolipids (Brown et al., 2019). Acute SGA treatment dramatically altered the homeostasis of central and peripheral sphingolipids (Castillo et al., 2016; Weston-Green et al., 2018). Notably, sphingolipids from bacteria were incorporated into the mammalian sphingolipid pathway (Johnson et al., 2020). The probiotic *Bacteroides* has also been shown to produce the endothelin-like molecule N-acyl-3-hydroxypalmitoyl-glycine (commendamide) (Cohen et al., 2015; Lynch et al., 2017). Furthermore, olanzapine-induced metabolic effects have been shown to be dependent on the endogenous cannabinoid system (Abolghasemi et al., 2021). Everard et al. showed that treating obese mice with *A. muciniphila* increased intestinal 2-oleoylglycerol (2-OG), 2-arachidonoylglycerol (2-AG) and 2-palmitoylglycerol (2-PG) levels (Everard et al., 2013). However, a recent study reported that *A. muciniphila* exerted its beneficial effects on metabolism independent of general changes in plasma endocannabinoidome mediators (Depommier et al., 2021). The gut microbiota–endocannabinoid axis is a key topic in the studies listed above, and it is likely to be a new target for SGA-induced lipid metabolism disorders.

### Central mechanism

Current evidence suggests that hyperphagic effects are responsible for a large percentage of the observed aberrations in lipid profiles, and there is a lack of satiety in both human and animal models in the presence of SGAs (Hartfield et al., 2003; Huang et al., 2020). The diversity of the gut flora is vital for appetite and metabolism regulation. Different gut bacteria and metabolites influence the gut’s ability to perceive nutrients, influencing the host’s appetite and energy metabolism (Oliphant and Allen-Vercoe, 2019). This is where the gut–brain axis becomes active. The gut–brain axis is a fundamental mechanism that links biochemical signals from the gastrointestinal tract to brain function (Carabotti et al., 2015). A sophisticated network of neurons regulates energy homeostasis in the host. Two types of neurons are particularly important for appetite control: neurons that express the neuropeptide proopiomelanocortin (POMC) (Baldini and Phelan, 2019) and those that express neuropeptide Y/agouti-related peptide (NPY/AgRP) (Han et al., 2018). These neurons interact with each other to form a switch that instantly adjusts appetite (Quarta et al., 2021). Among them, POMC neurons promote satiety, while AgRP neurons increase appetite.

On the one hand, gastrointestinal hormones affect the balance of the POMC/AgRP system, which controls appetite. Leptin (Endomba et al., 2020), CCK (Fan et al., 2004), PYY (Loh et al., 2015), GLP-1 (Teff et al., 2013), and 5-HT (Sohn et al., 2011; Bonn et al., 2013) activate POMC neuronal activity *via* receptors in the hypothalamus and inhibit NPY in AgRP neurons, sending appetite suppressant signals and regulating energy homeostasis and metabolism. Ghrelin is the only known gut hormone that promotes appetite by directly activating AgRP neurons and increasing the inhibitory effect of AgRP neurons on POMC neurons (Lage et al., 2010; Varela et al., 2011). On the other hand, the intestinal flora metabolites SCFAs and BAs can also influence appetite *via* the gut–brain axis. An increase in acetate production activates the parasympathetic nervous system, leading to an increase in gastrin secretion, which promotes host appetite (Perry et al., 2016). SCFAs also have the potential to enter the circulation, cross the blood‒brain barrier, and directly affect the central nervous system (Morrison and Preston, 2016). In addition, BAs can reach the hypothalamus and are highly correlated with circulating BA levels, which can reach the hypothalamus *via* passive diffusion, causing a brief increase in hypothalamic BA concentrations and triggering the expression of the AgRP/NPY neuronal membrane receptor TGR5, which in turn regulates appetite (Perino et al., 2021). It is worth noting that an imbalance in the amount of proinflammatory pathogenic bacteria can compromise intestinal wall integrity, affecting brain–gut axis transmission (Küme et al., 2017; Tilg et al., 2020). A study showed that the mRNA levels of NPY and AgRP were significantly increased in the hypothalamus of olanzapine-administered rats and were considerably lower than those in normal animals (Zhu Z et al., 2022). Some notable causes include several of these mechanisms affecting neuronal function through the gut–brain axis, which leads to hyperphagia and results in abnormal lipid profiles. Interestingly, this study showed that olanzapine-induced increases in weight gain percentage (WG%) occurred only when the vagus nerve was intact, while the negative effects of olanzapine-induced increases in white adipose tissue percentage (WAT%) and decreases in brown adipose tissue percentage (BAT%) were reversed by the disruption of the gut microbiota–brain axis (vagotomy), suggesting that an intact gut microbiota–brain axis may be necessary for olanzapine-induced disruption of lipid metabolism.

Lipopolysaccharide (LPS), a component of the outer membrane of most Gram-negative bacteria, is released upon bacterial cell death and enters the circulation through a ‟leaky gut”, resulting in increased levels of LPS in the blood (known as endotoxemia, which is a leading cause of metabolic diseases, such as insulin resistance, and is promoted by increased IL-6 and tumor necrosis factor (TNF) (Tilg et al., 2020)), which acts as a powerful stimulator of host immunity (Park and Lee, 2013). LPS is detected by Toll-like receptor 4 (TLR4) on the immune cell surface, resulting in the release of numerous cytokines and chemokines (Rhee, 2014). LPS can also interact directly with lipid molecules. All lipoproteins can bind to LPS and neutralize its toxicity *in vitro* and *in vivo* (Barcia and Harris, 2005).

### Peripheral tissue

#### SCFAs

SCFAs are used as a carbon source for the production of important endogenous host metabolites, such as fat and cholesterol (Besten et al., 2013). SCFAs produced by the intestinal flora are rapidly absorbed by colonic cells, due in part to monocarboxylate transporters, including the proton-coupled monocarboxylate transporter 1 (MCT1) and sodium-coupled monocarboxylate transporter 1 (SMCT1) (Dalile et al., 2019). The principal substrates for lipid synthesis in rat colonic epithelial cells, which convert SCFAs to acetyl coenzyme A (CoA), are acetate and butyrate (Zambell et al., 2003). CoA generates energy through the tricarboxylic acid cycle and produces palmitic acid under the action of the cytoplasmic enzyme system, which can be transferred to mitochondria to lengthen the carbon chain and form triglycerides with other substances stored in adipose tissue. In contrast, SCFAs that are not digested in colon cells enter the portal circulation of the liver through the basolateral membrane and provide substrates for hepatocyte energy metabolism. Carbohydrate-responsive element-binding protein (ChREBP) plays a key role in this process (Iizuka et al., 2020). A member of the acetyl-CoA synthetase short-chain family, encoded by Acss2, is induced by ChREBP and converts acetate to acetyl-CoA, which is used as a substrate for lipogenesis (BERG, 1956). Thus, regulating lipogenic gene expression and hepatic acetyl-CoA production from gut microbial acetate by inhibiting hepatic ChREBP is expected to prevent SGA-induced TG accumulation by inhibiting lipogenic gene expression and hepatic acetyl-CoA production. SCFAs are also involved in the biosynthesis of cholesterol and fatty acids in hepatocytes (Dalile et al., 2019). Chen et al. performed radiolabeling studies and showed that acetate was involved in the increase in *de novo* fat synthesis. Furthermore, antibiotic-treated mice showed reduced *de novo* fat synthesis (Kindt et al., 2018).

In addition, SCFAs are signaling molecules that regulate host-related functions mainly through two signaling pathways: the HDAC and G protein-coupled receptor signaling pathways. SCFAs have been shown to bind to the G protein-coupled receptors GPR43/FFAR2 and Gpr41/FFAR3 (Kimura et al., 2020), leading to further activation of downstream signaling cascades, including the phospholipase C (PLC), mitogen-activated protein kinase (MAPK), phospholipase A2 (PLA2) and nuclear factor-κB (NF-κB) pathways. Acetate inhibits insulin-mediated fat accumulation and improves lipid and glucose metabolism *via* GPR43. Mice lacking GPR43 were obese on a normal diet, whereas mice specifically overexpressing GPR43 in adipose tissue remained lean even when fed a high-fat diet. Both types of mice recovered under sterile conditions or after being treated with antibiotics (Kimura et al., 2013). GPR41 has been shown to regulate host energy homeostasis in a gut microbiota–dependent manner. Mice with knockout of the GPR41 gene exhibited a leaner body weight, but this difference was not observed in GF mice (Samuel et al., 2008). SCFAs also activate AMP-activated protein kinase (AMPK), a downstream signal of the G-protein-coupled receptor signaling pathway, and AMPK activation increases peroxisome proliferator-activated receptor-γ coactivator 1α (PGC-1α) expression in adipose tissue and skeletal muscle (Taylor et al., 2005; Wan et al., 2014; Yan et al., 2016). In addition, PGC-1α regulates the transcriptional activity of peroxisome proliferator-activated receptor α (PPARα) and peroxisome proliferator-activated receptor γ (PPARγ) (Muoio et al., 2002; Lin et al., 2005). Butyrate and propionate can activate PPARγ (Alex et al., 2013). Activation of liver and adipose tissue PPARγ by SCFAs regulates lipid metabolism by increasing energy expenditure, reducing inflammation in adipose tissue, improving insulin sensitivity, reducing body weight, and decreasing hepatic TG accumulation (Besten et al., 2015). A study in fish showed that the effects of olanzapine on lipid metabolism may be related to the regulation of the gut microbiota–SCFA–PPAR signaling pathway (Chang et al., 2022). The gut microbiome was significantly altered in carp that were administered olanzapine, as evidenced by an increase in the abundance of SCFA-producing bacteria, which led to an increase in the production of SCFAs. In addition, many genes that are components of the PPAR signaling pathway were significantly altered; specifically, the mRNA levels of genes related to lipid synthesis (including PPARγ, fatty acid synthase (FAS), and SREBP1) were significantly increased, and lipolysis-related genes (such as hormone-sensitive lipase (HSL) and PPARα) were significantly decreased. The activated AMPK signaling pathway can also promote the expression of HSL and adipose triglyceride lipase (ATGL), which promote lipolysis (Cantó and Auwerx, 2010; Deng et al., 2020; Guo et al., 2020; Tang et al., 2020). Jocken et al. performed *in vitro* experiments with a human white adipocyte model (human multipotent adipose tissue-derived stem (hMADS) cells). Acetate was found to be the main driver of the antilipolytic effect of SCFAs and attenuated HSL phosphorylation in hMADS adipocytes in a Gi-coupled manner (Jocken et al., 2017). This is reminiscent of the fact that the effect of SGAs on AMPK may also be an indirect consequence of the activation of AMPK by SCFAs in peripheral tissues. Indeed, olanzapine can reduce AMPK phosphorylation and activation in hepatocytes and 3T3-L1 cells, accompanied by a concomitant increase in SREBP-dependent lipid synthesis (Oh et al., 2011; Li et al., 2016). Interestingly, acetate supplementation did not attenuate olanzapine-induced weight gain in mice but appeared to increase it (Kao et al., 2019a). This concept of SCFA-induced weight gain appears to be consistent with the olanzapine-induced increase in plasma acetate (Kao et al., 2018).

#### BAs

BAs bind to FXR and TGR5 in the host and regulate lipid and energy metabolism (Chiang and Ferrell, 2019). FXR is a transcription factor that binds to the promoter region and induces the expression of multiple target genes and is expressed in the liver, ileum, kidney, and other tissues (Lefebvre et al., 2009; Teodoro et al., 2011). The most potent ligand for FXR is chenodeoxycholic acid (CDCA), followed by cholic acid (CA), deoxycholic acid (DCA), and lithocholic acid (LCA), all of which are FXR agonists. CDCA is converted to ursodeoxycholic acid in humans through a sequence of processes, and it does not activate FXR but rather inhibits FXR activity (Wang et al., 1999; Mueller et al., 2015). In addition, Sayin et al. identified two natural FXR antagonists: the taurine-conjugated murine BAs tauro-α-muricholic acid (TαMCA) and tauro-β-muricholic acid (TβMCA) (Sayin et al., 2013). TGR5 is a binding G-protein-coupled receptor expressed in tissues such as the intestine, liver, and brown‒white adipose tissue. TGR5 is mainly activated by the secondary BAs LCA and DCA (Maruyama et al., 2002; Kawamata et al., 2003).

FXR-deficient animals had increased hepatic and serum TG and cholesterol levels (Sinal et al., 2000). This finding indicates that FXR is required for lipid metabolism and energy homeostasis (Trauner et al., 2010). Reduced sterol-response element-binding protein-1c (SREBP-1c) expression caused by natural or synthetic FXR agonists *via* the FXR-SHP (small heterodimer partner) pathway could explain the inhibitory effect of BAs on TG production (Watanabe et al., 2004). In addition, Caron et al. used immortalized human hepatocyte (IHH) and HepaRG cell lines, which are glucose-responsive human hepatocyte lines, to show that the activation of FXR inhibits the transcriptional activity of ChREBP in human hepatocytes (Caron et al., 2013). BAs can also induce the expression of the human PPARα gene, which is a nuclear receptor that controls lipid and glucose metabolism and exerts anti-inflammatory effects *via* FXR (Pineda Torra et al., 2003). The activation of FXR has been shown to induce a decrease in serum apolipoprotein (Apo) CIII concentrations, leading to the amelioration of TG-rich remnant lipoprotein metabolism to reduce serum TG levels and cardiovascular risk profiles (Claudel et al., 2003). The FXR signaling pathway in mice and humans is significantly affected by SGAs. At present, pharmacological therapies that target FXR in combination with SGAs are still needed to translate the positive findings of these studies into practical outcomes. Exposure of a mouse precision-cut liver slice (PCLS) model to chlorpromazine significantly altered cholesterol and BA cellular transport regulated by FXR and BA regulation of glucose and lipid metabolism *via* FXR (Szalowska et al., 2013). In addition, a study also showed the downregulation of FXR targets such as Bsep, Mdr3, Ntcp, and Cyp8b1. This finding was consistent with that observed in chlorpromazine-treated HepaRG cells (Anthérieu et al., 2013). As a next step, more experiments on the effect of SGAs on FXR are needed to further understand the beneficial effects of chlorpromazine.

TGR5 has also been shown to be a BA-responsive receptor involved in host lipid metabolism. In muscle and brown adipose tissue, TGR5 may play a role in energy homeostasis by promoting intracellular thyroid hormone activity and thereby increasing energy expenditure (Watanabe et al., 2006). In addition, TGR5 has been shown to activate PPARα and PGC-1α to increase mitochondrial oxidative phosphorylation and energy metabolism (Chiang and Ferrell, 2020). However, there are limited data on changes in TGR5 receptor activity in schizophrenia patients during the use of SGAs.

#### GLP-1

Ishøy et al. published the first clinical data supporting the use of the GLP-1 agonist liraglutide to treat clozapine-induced lipid profile disturbances and weight gain in schizophrenia (Ishøy et al., 2013). Consistent with this study, Larsen et al. and Siskind et al. demonstrated that GLP-1 agonists could be effective in reducing clozapine- or olanzapine-induced lipid metabolism disorders (Kouidrat and Amad, 2019). GLP-1, a glucose-dependent incretin, plays a crucial role in lipid metabolism and body weight maintenance by binding to the GLP-1 receptor (GLP-1R). Many human tissues, including the pancreas, liver, muscle, fat, gastrointestinal tract, heart, and brain, express GLP-1R (Campbell and Drucker, 2013). When bound to GLP-1, GLP-1R acts through its coupled G protein (Gαs) in pancreatic β-cells, activating adenylyl cyclase and increasing the intracellular levels of cyclic adenosine monophosphate (cAMP) (Doyle and Egan, 2007); this increase in cAMP exerts a series of effects. The activation of factors (protein kinase A (PKA) (Béguin et al., 1999) and exchange protein directly activated by cAMP (EPAC) (Kang et al., 2008)) leads to calcium influx, increased transcription of the proinsulin gene, and the stimulation of insulin secretion. In addition, GLP-1R may regulate pancreatic β-cell metabolism by activating the phosphoinositide 3-kinase (PI3K)/AKT (protein kinase B)/mTOR (mammalian target of rapamycin) and MAPK signaling pathways (Rowlands et al., 2018). The binding of GLP to GLP-1R in adipocytes activates the adenylyl cyclase (AC)/cAMP signaling pathway, regulates the apoptosis and proliferation of preadipocytes through various cellular signaling pathways, such as extracellular signal–regulated kinase (ERK), protein kinase C (PKC), and AKT, and alters the expression of PPARγ and its target genes (Challa et al., 2012; Chen et al., 2017). Furthermore, by reducing macrophage infiltration in adipose tissue, GLP-1 can directly block the inflammatory signaling pathway, improving insulin resistance, lowering liver fat levels, and considerably alleviating NAFLD (Blaslov et al., 2014). This explains how GLP-1R might decrease hepatic substrate supply (e.g., glucose and non-esterified fatty acids (NEFAs)) by affecting adipose tissue, which may be partially responsible for the overall effect. GLP-1R-based treatment of metabolic diseases has been reported to act on hepatocyte lipid metabolism through PI3K, type 1 protein phosphatase (PP-1), and PKC (Redondo et al., 2003). Interestingly, a study showed that liraglutide ameliorated hepatocyte steatosis by inducing autophagy through the AMPK/mTOR pathway (He et al., 2016). Additionally, GLP-1 may promote hepatocyte survival by downregulating microRNA-23, resulting in increased expression of PGC-1α and uncoupling protein 2 (UCP2) (Wang C et al., 2015). Recent studies have shown that GLP1/GLP-1R signaling is involved in the effect of brexpiprazole, a new multitarget antipsychotic drug (APD) approved by the US FDA in 2015 that induces disorders of glucose and lipid metabolism (Li et al., 2021). Brexpiprazole administration significantly reduced the protein and mRNA levels of GLP1 in the pancreas and small intestine by inhibiting Ca^2+^/calmodulin-dependent kinase IIα (CaMKIIα), AMPK, and β-catenin. Brexpiprazole administration also caused islet dysfunction and decreased GLP-1R, PI3K, and IRβ expression in the pancreas. Cotreatment with liraglutide and brexpiprazole is an effective strategy for certain aberrant metabolisms.

#### Leptin

Leptin was found to be involved in lipid metabolism and energy balance by mediating certain signaling pathways. Leptin inhibits acetyl-CoA carboxylase (ACC) activity by activating AMPK in skeletal muscle, thereby stimulating the oxidation of fatty acids (Minokoshi et al., 2002). Consistently, another study revealed that the activation of AMPK can have a therapeutic effect on metabolic syndrome only if leptin is present and active (Stockebrand et al., 2013). In addition, p38 MAPK may also contribute to the effect of leptin on fatty acid oxidation (Dardeno et al., 2010). In non-adipose tissues, leptin may promote fatty acid oxidation by activating PPARα-induced CoA expression *via* signal transducer and activator of transcription 3 (STAT3) (Unger et al., 1999). Maya et al. found that leptin could regulate lipid metabolism and inflammation by modulating the PI3K/Akt/mTOR pathway (Maya-Monteiro and Bozza, 2008). Consistent with this report, Schmidt et al. found that olanzapine simultaneously upregulated the mTOR pathway and downstream signaling cascades, including the activation of mTORC1, in mice (Schmidt et al., 2013). mTORC1 activation interferes with lipid and energy metabolism, leading to the upregulation of lipid biosynthesis and the accumulation of TGs. Furthermore, activation of the mTOR pathway inhibits autophagy, thereby increasing intracellular lipid accumulation (Zhuo et al., 2022). Enhanced mTOR activity disrupts hepatic lipid homeostasis by regulating the expression of the transcription factor SREBP-1c (Takashima et al., 2009).

## Strategies for modifying the gut microbiome to ameliorate SGA-induced disorders of lipid metabolism

### Pharmacological interventions

To date, the mechanisms of SGA-induced metabolic changes have not been thoroughly investigated. However, in clinical treatment, side effects of SGAs on lipid metabolism can usually be suppressed by other drugs, and some interventions have yielded significant results. Researchers found that metformin, a biguanide antihyperglycemic agent, had a positive effect on the lipid profile, insulin resistance, and body weight in patients with schizophrenia, which has been supported by animal (Zhu W et al., 2022) and human models (Wu et al., 2016; Vancampfort et al., 2019; Jiang et al., 2020). Interestingly, the intestinal flora plays a vital role in the positive effects of metformin. Luo et al. (Luo et al., 2021) and Wang et al. (Wang et al., 2021) found that metformin not only prevented olanzapine-induced disruption of the lipid profile and hepatic histopathological changes but also partially reversed olanzapine-induced alterations in the gut microbiota and helped correct peripheral and central satiety-related neuropeptide disorders. This finding demonstrated that the gut–brain axis is a mediator by which metformin ameliorates SGA-induced metabolic dysfunction. Statins are also considered a potential preventive and therapeutic approach to reduce SGA-induced weight gain and dyslipidemia in patients with schizophrenia. It has been reported that pravastatin (Vincenzi et al., 2014), atorvastatin (Ojala et al., 2008), lovastatin (Ghanizadeh et al., 2014), rosuvastatin (Hert et al., 2006), or simvastatin (Tajik-Esmaeeli et al., 2017) in combination with SGAs can reduce TC, LDL cholesterol, and TG levels in patients with schizophrenia. Animal studies have shown that statins improve SGA-induced metabolic disturbances partly due to statin-mediated modulation of BAT activity (Liu et al., 2020) and inhibition of the hepatic mTOR signaling pathway (Liu et al., 2019). Interestingly, statins were also recently shown to improve the gut microbiota, which seems to partially explain the associated clinical improvements (Kim et al., 2019; Vieira-Silva et al., 2020).

### Non-pharmacological interventions

New biological therapeutic strategies, including probiotics, prebiotics, gut hormone, and fecal microbiota transplantation (FMT), are being explored to directly target the gut microbiota and its metabolite products to improve SGA-induced dyslipidemia. Probiotics have been shown to play a vital role in lipid homeostasis in the host (Table 3). However, there have been few studies on the effects of probiotics and prebiotics on SGA-induced changes in lipid metabolism and energy. Tomasik et al. discovered that probiotics and prebiotics could alleviate SGA-induced gastrointestinal distress (Tomasik et al., 2015). However, the effects of probiotics and prebiotics on SGA-induced changes in lipid metabolism are unclear and controversial because the effects of these factors on lipid metabolism are strain and population specific. For example, the probiotic *A. muciniphila* (Huang et al., 2021) or prebiotic B-GOS (Kao et al., 2018) can partially reverse olanzapine-induced disturbances in the gut microbiota and lipid metabolism in rats. The probiotic mixture VSL#3, a mixture of eight different bacterial probiotic species, was shown to attenuate olanzapine-induced body weight gain, uterine fat deposition, and dyslipidemia (Dhaliwal et al., 2019). Importantly, while their effectiveness has been relatively well documented in animal studies, translation to humans has sometimes shown controversy. Kao et al. found that B-GOS supplementation did not affect SGA-induced weight gain or changes in circulating metabolic markers, contrary to their observations in rats (Kao et al., 2019b). Yang et al. reported that the addition of probiotics, including *Bifidobacterium* and *Lactobacillus*, was not sufficient to reduce weight gain in patients with schizophrenia, nor did it significantly improve lipid profiles (Yang et al., 2021). In comparison, the combined use of probiotics and dietary fiber was effective in reducing olanzapine-induced weight gain without any apparent adverse effects while maintaining the desired psychopathological effect (Liu et al., 2021; Huang et al., 2022a; Huang et al., 2022b). Therefore, more randomized controlled trials in humans are needed to translate beneficial findings in animals. Indeed, *A. muciniphila* has been shown to be safe and effective in human trials, and pasteurized *A. muciniphila* is more effective than live *A. muciniphila* (Depommier et al., 2019). In addition, the gut hormone GLP-1 has demonstrated the potential to improve SGA-induced disorders of lipid metabolism. The combination of liraglutide, a GLP-1 receptor agonist, and SGAs has potential benefits on body weight and lipid metabolism in patients with schizophrenia, but patients must receive daily subcutaneous injections and have a relatively high rate of adverse events (Whicher et al., 2019). In contrast, FMT, which is being researched as an alternative to SGAs (Settanni et al., 2021), lacks experimental data to demonstrate its potential in SGA-induced metabolic disorders.

**TABLE 3:** Effects of prebiotic supplementation on host lipid metabolism.

Subject	Models	Probiotics	Implicated microbiota	Changes in lipid profile			References
				Serum	Liver	Fecal	
Sprague-Dawley rats	HCD	*Lactobacillus* plantarum MA2	↑: Lactic acid bacteria and Bifidobacterium	↓: TC, LDL-C, and TG	↓: TC and TG	↑: TC and TG	Wang et al. (2009)
Sprague-Dawley rats	HCD	*Lactobacillus* acidophilus 4356	↑: Lactobacilli and Bifidobacteria	↓: TC, LDL-C, TAG	↓: TC and TAG		Huang et al. (2010)
Sprague-Dawley rats	HFD	Bifidobacteria L66–5, L75–4, M13-4 and FS31-12		↓: TC and TG	↓: TC and TG		Yin et al. (2010)
Sprague-Dawley rats	HCD	*Lactobacillus* plantarum 9–41-A, *Lactobacillus* fermentum M1-16	↑: *Lactobacillus* and Bifidobacterium	↓: TC, LDL-C, TG	↓: TC and TG	↓: TC	Xie et al. (2011)
Sprague-Dawley rats	HFD	Bifidobacterium pseudocatenulatum SPM 1204, Bifidobacterium longum SPM 1205, and Bifidobacterium longum SPM 1207	↑: Lactobacilli	↓: TC, LDL-C, HDL-C, and TG			An et al. (2011)
Wistar rats	HFD	Lactobacilli LIP-1, MG9-2, and E7301		↑: HDL-C; ↓: TC, LDL-C, and TG	↓: TG	↑: TC	Wang et al. (2012)
Sprague-Dawley rats	HFD	*Lactobacillus* plantarum LS/07, *Lactobacillus* plantarum Biocenol LP96		L. LS/07; ↓: TC and LDL-C; L.LP96; ↓: TG and VLDL	no significant change	↑: TC and TG	Salaj et al. (2013)
Sprague-Dawley rats	HCD	*Lactobacillus* plantarum NS5, *Lactobacillus* delbrueckii subsp	↑: *Bacteroides*; ↓: Clostridium	↑: ApoA-I; ↓: TC, HDL-C, Apo-B, FFAs	↓: TC and TG		Hu et al. (2013)
C57BL/6J mice	HFD + HCD	*Lactobacillus* plantarum KY1032, *Lactobacillus* curvatus HY7601		L. KY1032; ↑: TG; L. HY7601; ↑: TG; ↓: TC; L. KY1032 + L. HY7601; ↑: TG; ↓: TC and LDL-C	L. HY7601; ↓: TC, TG, and FFAs; L. KY1032 + L. HY7601; ↓: TC, TG, and FFAs	↑: TC and TG	Yoo et al. (2013)
C57BL/6J mice	HFD	*Lactobacillus* curvatus HY7601 and *Lactobacillus* plantarum KY1032	↓: Proteobacteria	↓: TC			Park et al. (2013)
C57BL/6J mice	HFD	*Lactobacillus* rhamnosus GG		ORO staining	ORO staining		Kim et al. (2013)
Human	overweight children	Synbiotic capsules	↑: *Lactobacillus*	↓: TC, LDL-C, and TG			Safavi et al. (2013)
C57BL/6J mice	HFD	*Lactobacillus* casei NCDC 19		↓: TC and LDL-C			Rather et al. (2014)
Albino rats	HCD	*Lactobacillus* reuteri LR6		↓: TC, LDL, TG			Singh et al. (2015)
Wistar rats	HCD	Kluyveromyces marxianus M3		↑: HDL-C; ↓: TC, LDL-C, and TG	↑: HDL-C; ↓: TC, LDL-C, and TG		Xie et al. (2015)
C57BL/6J mice	HFD	*Lactobacillus* paracasei CNCM I-4270, L. rhamnosus I-3690 and Bifidobacterium animalis subsp. lactis I-2494	↑: *Lactobacillus* paracasei CNCM I-4270, L. rhamnosus I-3690 and Bifidobacterium	HE staining	HE staining		Wang J et al. (2015)
Human	overweight adults	*Lactobacillus* curvatus HY7601 and *Lactobacillus* plantarum KY1032		↑: ox-LDL			Jung et al. (2015)
Sprague-Dawley rats	HCD	*Lactobacillus* plantarum Lp3	↑: *Lactobacillus* and Bifidobacterium; ↓: Escherichia coli	↓: TC, LDL-C, and TG	↓: TC and TG	↑: TC and TBA	Ding et al. (2017)
Wistar rats	HFD	*Lactobacillus* plantarum YS5		↑: HDL-C; ↓: TC, LDL-C, and TG			Nami et al. (2019)
Wistar rats	HCD	*Lactobacillus* fermentum PD2 and PH5	↑: *Lactobacillus*; ↓: coliforms	↓: TC, LDL-C, and TG	↓: TC		Thakkar et al. (2020)
Sprague-Dawley rats	HFD	*Lactobacillus* plantarum LS/07	↑: Lactobacilli; ↓: Coliforms	↓: TC, LDL-C, ox-LDL			Hijova et al. (2020)
Human	hypercholesterolemia	Lactoplantibacillus plantarum strains (CECT7527, CECT7528, and CECT7529)		↓: TC and LDL-C			Guerrero-Bonmatty et al. (2021)
Human	overweight adults	*Lactobacillus* plantarum K50	↑: *Lactobacillus* plantarum; ↓: Actinobacteria	↓: TC and TG			Sohn et al. (2021)
Human	overweight adult women	Bifidobacterium lactis UBBLa-70		↑: arginine, glutamine, and 2-oxoisovalerate; ↓: glycerol			Crovesy et al. (2021)

HCD, high cholesterol diet; HFD, high fat diet; TC, total cholesterol; TG, triglycerides; LDL-C, low density liptein cholesterol; HDL-C, high density liptein cholesterol; ox-LDL, oxidized low-density lipoprotein; LDL, low-density lipoprotein; TBA, total bile acids; TAG, triacylglycerols; VLDL, very-low-density lipoprotein; ORO, staining, Oil Red O staining; HE, staining, hematoxylin-eosin staining; Apo-B, apolipoprotein B; ApoA-I, apolipoprotein A-I; FFAs, Free fat acids.

## Future perspectives

**FIGURE 2:**
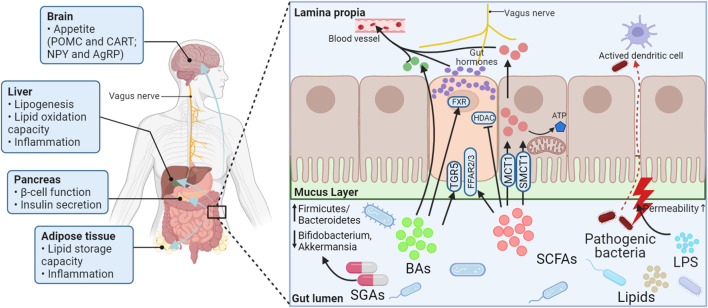
Schematic presentation of the potential mechanism of lipid metabolism disorders secondary to SGA treatment based on the gut microbiota. Treatment with SGAs may increase the relative ratio of Firmicutes to Bacteroidetes bacteria, As well as decrease the relative abundance of Bifidobacterium and Akkermansia muciniphila. The products of the gut microbiota (lipids, LPS, SCFAs, and BAs) change as a result of this transformation. SCFAs activate FFAR2/3 or HDAC. BAs send signals to EE cells through TGR5 or nuclear FXR, allowing EE cells to synthesize and secrete various gut hormones. LPS, SCFAs, BAs, and gut hormones are important players in interorgan crosstalk by affecting appetite, regulating gut integrity, and improving liver, pancreas, and adipose tissue function and lipid metabolism. POMC, Proopiomelanocortin; CART, Cocaine- and amphetamine-regulated transcript; NPY, Neuropeptide Y; AgRP, Agouti-related peptide; FXR, Farnesoid X receptor; HDAC, Histone deacetylase; ATP, Adenosine triphosphate; TGR5, Takeda G protein-coupled receptor 5; FFAR2/3, Free fatty acid receptors 2/3; MCT1, Proton-coupled monocarboxylate transporters 1; SMCT1, Sodium-coupled monocarboxylate transporters 1; BAs, Bile acids; SCFAs, Short-chain fatty acids; LPS, Lipopolysaccharide; SGAs, Second-generation antipsychotics.

**FIGURE 3:**
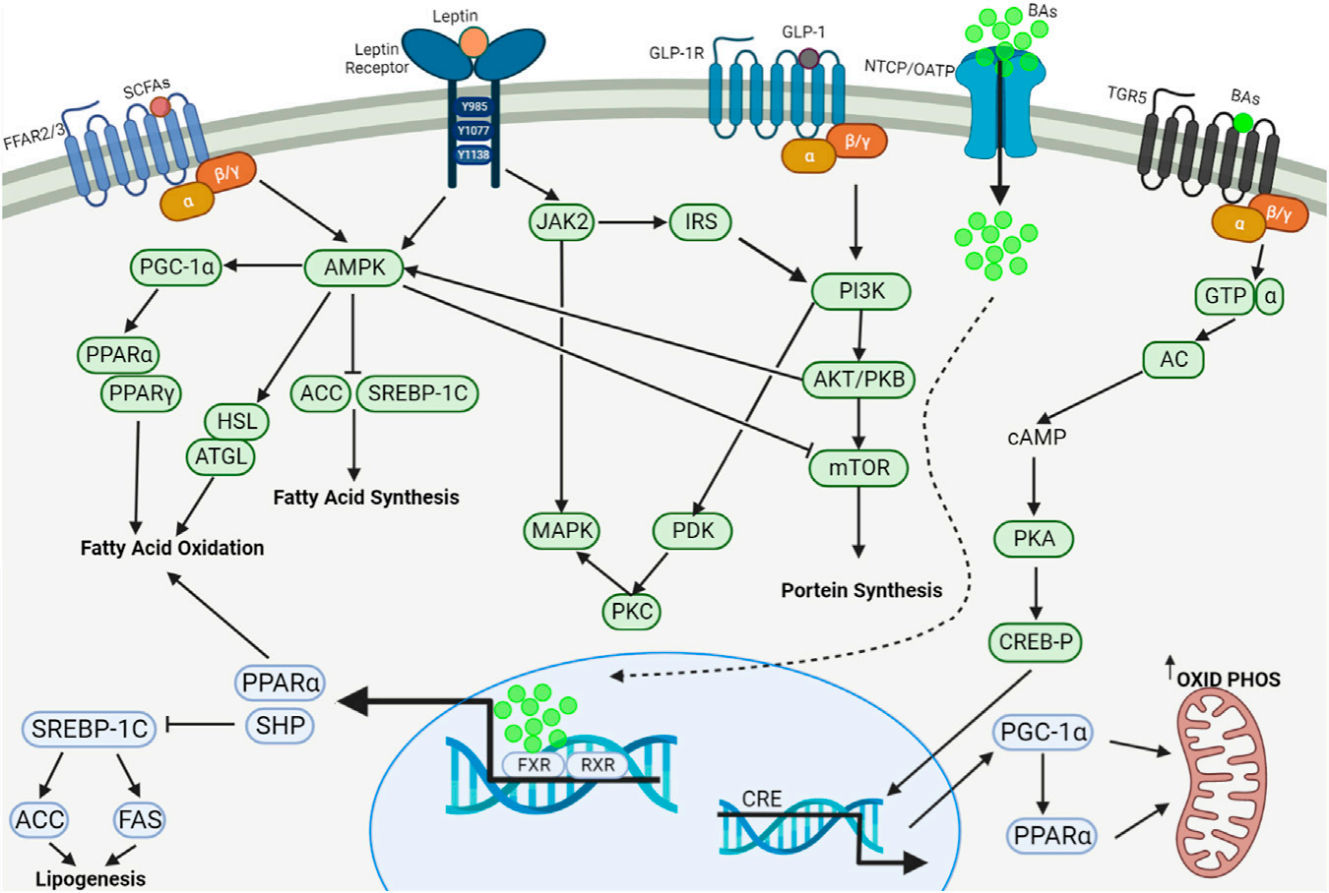
Regulation of lipid metabolism by SCFAs, BAs, leptin and GLP-1. The major signaling pathways including AMPK, MAPK, PI3K/Akt/mTOR and cAMP/PKA/CREB-P work systematically in concert to regulate fatty acid oxidation, fatty acid synthesis, protein synthesis and mitochondrial oxidative phosphorylation and energy metabolism. SCFAs: Short-chain fatty acids; GLP-1: Glucagon-like peptide 1; BAs, Bile acids; FFAR2/3, Free fatty acid receptors 2/3; GLP-1R, GLP-1 receptor; NTCP, Na^+^-taurocholate cotransporting polypeptide; OATP, Organic anion transporting polypeptide; TGR5, Takeda G protein-coupled receptor 5; AMPK, AMP-activated protein kinase; PGC-1α, Peroxisome proliferator-activated receptor-γ coactivator 1α; PPARα, Peroxisome proliferator-activated receptor α; PPARγ, Peroxisome proliferator-activated receptor γ; HSL, Hormone-sensitive lipase; ATGL, Adipose triglyceride lipase; ACC, Acetyl coenzyme A carboxylase; SREBP-1c, Sterol response element-binding protein-1c; JAK2, Janus kinase-2; MAPK, Mitogen-activated protein kinase; IRS, Insulin receptor substrate; PDK, Phosphoinositide-dependent protein kinase; PKC, Protein kinase C; PI3K, Phosphoinositide 3-kinase; AKT, Protein kinase B (PKB); mTOR, Mammalian target of rapamycin; GTP, Guanosine triphosphate; AC, Adenyl cyclase; cAMP, Cyclic adenosine monophosphate; PKA, Protein kinase A; CREB-P, Phosphorylated CREB (cAMP-response element-binding protein); FXR, Farnesoid X receptor; RXR, Retinoid X receptor; CRE, cAMP response element; SHP, Small heterodimer partner; FAS, Fatty acid synthase.

Long-term use of SGAs can cause weight gain and increase lipids, which can lead to an increased chance of patients suffering from metabolic syndrome, thereby increasing the risk that they will develop hypertension and cardiovascular and cerebrovascular diseases. During this process, the microbiome is both essential and sufficient, and several pathways involved in lipid metabolism have been postulated (Figure 2). First, SGAs directly inhibit the growth of microbial species that produce specific lipids (e.g., endogenous cannabinoids, and cholesterol). Second, SCFAs and BAs produced by the gut microbiota can regulate gut hormones such as CCK, PYY, GLP-1, and 5-HT. On the one hand, these signaling molecules can stimulate the vagus nerve or be carried into the brain to affect appetite *via* the gut–brain axis; on the other hand, they can regulate lipid metabolism *via* peripheral signaling pathways (Figure 3).

However, many unanswered questions remain. Which components of the gut microbiota and host metabolism are chiefly associated with schizophrenia? Are the microbiota changes observed in schizophrenia treated with SGAs secondary to SGA treatment? What are the metabolic side effects of SGAs and their impact on the microbiota? Do changes in the gut microbiota affect the efficacy of SGAs? To answer these questions, further experimental data are needed. This will lead to improved schizophrenia treatment options, individualized therapy, and the prediction and mitigation of side effects.

Current strategies to modulate the gut microbiome to improve SGA-induced lipid metabolism disturbances are particularly promising. Prebiotics, probiotics, and FMT have achieved certain curative effects in animal experiments. As a next step, more randomized controlled trials into humans are needed to translate the beneficial findings in animals. It is important to observe changes in host lipid metabolism after concurrent administration of SGAs and the abovementioned treatments. Compared with prebiotic therapy and other drug interventions, probiotic treatment offers superior specificity and safety. To develop this specific microbial therapeutic approach, a better understanding of the precise role of microbes in SGA-related lipid metabolism and elucidation of the linkages between specific microbiota and lipid profiles of the gastrointestinal tract will be needed. Furthermore, proper exercise and diet must not be overlooked.
